# Supplementary material for: The genome sequence of the Maple Pug moth, Eupithecia inturbata (Hübner, 1817)
Source: Wellcome Open Res. 2025 Mar 18;10:141. [Version 1] doi: 10.12688/wellcomeopenres.23796.1 (PMC11992513; doi:10.12688/wellcomeopenres.23796.1)
Supplement: Peer review history

# Reviewer report: Reviewer response for version 1

**DOI:** 10.21956/wellcomeopenres.26246.r121415

**Published:** 2025-04-25

**Authors:** Maríndia Deprá

**Affiliations:** 1 Federal University of Rio Grande do Sul, Porto Alegre, Brazil

The paper titled “The genome sequence of the Maple Pug moth, Eupithecia inturbata (Hübner, 1817)” is a Data Note presenting the nuclear and mitochondrial genome assemblies of Eupithecia inturbata. The nuclear genome spans 427.76 Mb and has been scaffolded into 31 chromosomal pseudomolecules, representing 30 autosomes and the Z sex chromosome. The mitochondrial genome is 15.33 Kb in length.

 The methods employed follow standard practices for bioinformatics analyses, utilizing well-established pipelines such as Nextflow, BUSCO, and DIAMOND. The manuscript provides detailed descriptions of the software tools, databases, and parameters used, along with explanation of each step in the data processing workflow.

 Although the manuscript does not include direct links to the datasets, the accession numbers are provided, ensuring data accessibility and enabling potential reuse.

 Overall, the manuscript is well structured and technically sound. However, it would benefit from the inclusion of additional information about the species, such as aspects of its biology and habitat.

 One methodological point that could be clarified is the identification of the Z chromosome: the manuscript states that this was based on read coverage statistics, but it is unclear which metrics were used. Wouldn’t homology-based identification be more appropriate? Furthermore, identification of the W chromosome would also be desirable.

 Finally, I suggest revising the sections that refer to the sex of the sequenced specimen. In some parts of the manuscript (e.g., the abstract and page 3), it is described as female, while in others it is referred to as male.

Are sufficient details of methods and materials provided to allow replication by others?

Yes

Is the rationale for creating the dataset(s) clearly described?

No

Are the datasets clearly presented in a useable and accessible format?

Yes

Are the protocols appropriate and is the work technically sound?

Yes

Reviewer Expertise:

genetics and genomics

I confirm that I have read this submission and believe that I have an appropriate level of expertise to confirm that it is of an acceptable scientific standard, however I have significant reservations, as outlined above.

# Reviewer report: Reviewer response for version 1

**DOI:** 10.21956/wellcomeopenres.26246.r121412

**Published:** 2025-04-11

**Authors:** Panagiotis Ioannidis

**Affiliations:** 1 Foundation for Research & Technology - Hellas, Crete, Greece

The present manuscript presents the genome assembly and initial annotation of a lepidopteran species.

 I would like to see a more detailed overview of the biology of this insect in the Background section. Currently the authors only mention the site from where they collected the sample.

 Other than this the manuscript is very well-written with many technical details explaining all the methodology. Moreover, the figure legends are among the best I've seen!

 Like other genome reports in this journal, a gene set was generated with the Ensembl Rapid Release pipeline. My first comment on this is that it would be nice for the reader to have at least a few details on the methodology followed for gene prediction (e.g. was RNAseq used as evidence, which proteins were used as evidence, etc). I guess that a detailed report can be found at Ensembl's web site, and that's why I'm not asking for details; a few basic things would be enough. My second comment is one that I have mentioned in my other reviews for this journal, but I should mention it again here; it is almost obligatory (!) to have the BUSCO scores for the generated gene set and put them side-by-side with the BUSCO scores from the corresponding genome. This way the reader can see how good is the gene prediction pipeline (normally, the gene set score should be higher than the genome score).

Are sufficient details of methods and materials provided to allow replication by others?

Yes

Is the rationale for creating the dataset(s) clearly described?

Yes

Are the datasets clearly presented in a useable and accessible format?

Yes

Are the protocols appropriate and is the work technically sound?

Yes

Reviewer Expertise:

bioinformatics, insect genomics, insect transcriptomics

I confirm that I have read this submission and believe that I have an appropriate level of expertise to confirm that it is of an acceptable scientific standard.

# Reviewer report: Reviewer response for version 1

**DOI:** 10.21956/wellcomeopenres.26246.r121416

**Published:** 2025-04-11

**Authors:** Annabel Whibley

**Affiliations:** 1 The University of Auckland, Auckland, New Zealand

This Data Note reports the genome assembly and Ensembl gene annotation of the Maple Pug moth Eupithecia inturbata. 

**Please note that there is an error in the methods: **the specimen is recorded as male in the “Sample acquisition” section. This should be corrected to avoid confusion.

 The assembly is of high quality, with clear reporting of metadata and methods and use of appropriate bioinformatics tools. The accession links are functional. The report follows DToL templating with  clear presentation of relevant information. 

 The apparent absence of the W chromosome from this female is interesting. The reduced dosage of the Z chromosome compared to autosomes is consistent with the female identification of the specimen. Atypical sex chromatin has been described in Geometrids, including in the family Larentiinae (e.g. Cells 2021, 10(9), 2230; https://doi.org/10.3390/cells10092230).  It would be interesting to explore this finding further (and to compare to closely related species).

Are sufficient details of methods and materials provided to allow replication by others?

Yes

Is the rationale for creating the dataset(s) clearly described?

Yes

Are the datasets clearly presented in a useable and accessible format?

Yes

Are the protocols appropriate and is the work technically sound?

Yes

Reviewer Expertise:

Genomics, Bioinformatics

I confirm that I have read this submission and believe that I have an appropriate level of expertise to confirm that it is of an acceptable scientific standard, however I have significant reservations, as outlined above.

# Reviewer report: Reviewer response for version 1

**DOI:** 10.21956/wellcomeopenres.26246.r120962

**Published:** 2025-04-05

**Authors:** Sivasankaran Kuppusamy

**Affiliations:** 1 Loyola College, Chennai, India

The authors have done chromosome-level genome assembly of the Maple Pug moth, *Eupithecia inturbata* (Hubner, 1817).  The species' genome size was estimated at 427.76 Mb. They have extracted and sequenced DNA using the correct methodology. Furthermore, they assembled and annotated using the appropriate software. The comprehensive data will be necessary for the phylogenomic studies of the lepidopteran moths.  

**Comments on the manuscript:**

- The first sentence of the Background can include the author’s name of the species. The sentence may change like this “The genome of the Maple Pug, *Eupithecia inturbata *(Hubner, 1817)”….
- The authors consistently included the complete name of the genus, *Eupithecia inturbata,* throughout the article. If at all possible, start with the full genus name and then abbreviate it to *E. inturbata.*
- Authors have mentioned the sequence was done based on a female specimen in the Abstract, Background and Genome sequence report. But in the Methods authors have mentioned “An adult male *E. inturbata* was collected from Wytham Woods”. I think there is an error. Authors can correct it. 

Above all, I confirm that the manuscript meets the necessary scientific standard and is suitable for indexing

Are sufficient details of methods and materials provided to allow replication by others?

Yes

Is the rationale for creating the dataset(s) clearly described?

Yes

Are the datasets clearly presented in a useable and accessible format?

Yes

Are the protocols appropriate and is the work technically sound?

Yes

Reviewer Expertise:

Phylogeny of Lepidoptera

I confirm that I have read this submission and believe that I have an appropriate level of expertise to confirm that it is of an acceptable scientific standard.
